# Ethyl 7-chloro-1-cyclo­propyl-6-fluoro-8-nitro-4-oxo-1,4-dihydro­quinoline-3-carboxyl­ate

**DOI:** 10.1107/S1600536812011373

**Published:** 2012-07-25

**Authors:** Raed A. Al-Qawasmeh

**Affiliations:** a Department of Chemistry, University of Jordan, Amman 11942, Jordan

## Abstract

In the title compound, C_15_H_12_ClFN_2_O_5_, mol­ecules are packed in the crystal lattice in a parallel fashion sustained by various C—H⋯O [C⋯O = 3.065 (5)–3.537 (5) Å] and C—H⋯Cl [3.431 (5)–3.735 (4) Å] inter­actions.

## Related literature

For the biological activities of fluoro­quinolone derivatives, see: Li *et al.* (2000 ▶); Mitscher (2005 ▶). For the synthesis of the title compound, see: Al-Qawasmeh *et al.* (2009 ▶); Al-Hiari *et al.* (2006 ▶). 
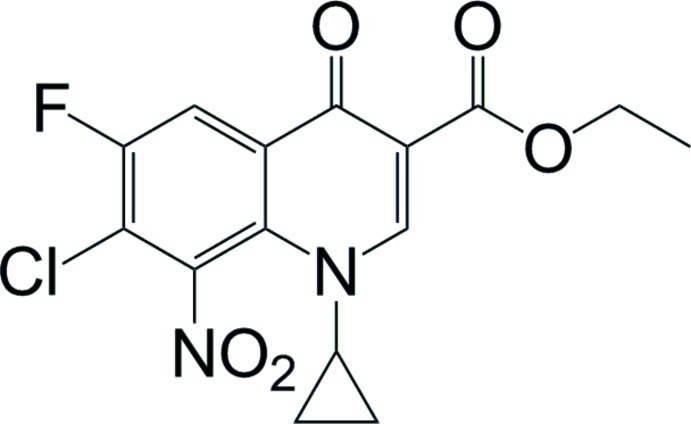


## Experimental

### 

#### Crystal data

C_15_H_12_ClFN_2_O_5_
*M* *_r_* = 354.72
Triclinic, 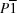

*a* = 8.2339 (16) Å
*b* = 9.1523 (18) Å
*c* = 10.736 (2) Å
α = 85.60 (3)°
β = 81.20 (3)°
γ = 74.13 (3)°
*V* = 768.5 (3) Å^3^
*Z* = 2
Mo *K*α radiation
μ = 0.29 mm^−1^
*T* = 291 K
0.96 × 0.35 × 0.21 mm

#### Data collection

Oxford Diffraction Xcalibur Eos diffractometer
Absorption correction: multi-scan (*CrysAlis PRO*; Oxford Diffraction, 2009 ▶) *T*_min_ = 0.857, *T*_max_ = 1.000
4468 measured reflections
2713 independent reflections
1617 reflections with *I* > 2σ(*I*)
*R*_int_ = 0.031

#### Refinement

*R*[*F* ^2^ > 2σ(*F* ^2^)] = 0.060
*wR*(*F* ^2^) = 0.195
*S* = 1.05
2713 reflections
218 parameters
H-atom parameters constrained
Δρ_max_ = 0.29 e Å^−3^
Δρ_min_ = −0.26 e Å^−3^

### 

Data collection: *CrysAlis PRO* (Oxford Diffraction, 2009 ▶); cell refinement: *CrysAlis PRO*; data reduction: *CrysAlis PRO*; program(s) used to solve structure: *SHELXS97* (Sheldrick, 2008 ▶); program(s) used to refine structure: *SHELXL97* (Sheldrick, 2008 ▶); molecular graphics: *OLEX2* (Dolomanov *et al.*, 2009 ▶); software used to prepare material for publication: *SHELXL97*.

## Supplementary Material

Crystal structure: contains datablock(s) I, global. DOI: 10.1107/S1600536812011373/ds2180sup1.cif

Structure factors: contains datablock(s) I. DOI: 10.1107/S1600536812011373/ds2180Isup2.hkl

Supplementary material file. DOI: 10.1107/S1600536812011373/ds2180Isup3.cml

Additional supplementary materials:  crystallographic information; 3D view; checkCIF report

## Figures and Tables

**Table 1:** Hydrogen-bond geometry (Å, °)

*D*—H⋯*A*	*D*—H	H⋯*A*	*D*⋯*A*	*D*—H⋯*A*
C14—H14*A*⋯O1^i^	0.97	2.54	3.489 (4)	167
C14—H14*B*⋯O1^ii^	0.97	2.51	3.471 (5)	172
C15—H15*A*⋯O2^iii^	0.98	2.58	3.537 (5)	165
C4—H4*A*⋯O2^iv^	0.93	2.71	3.065 (5)	104
C13—H13*A*⋯O4^ii^	0.97	2.71	3.439 (5)	132
C11—H11*A*⋯Cl1^v^	0.97	2.91	3.431 (5)	115
C13—H13*A*⋯Cl1^vi^	0.97	2.89	3.735 (4)	146

Symmetry codes: (i) 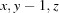; (ii) 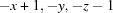; (iii) 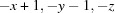; (iv) 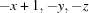; (v) 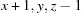; (vi) 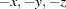.

**Fig. 1.**
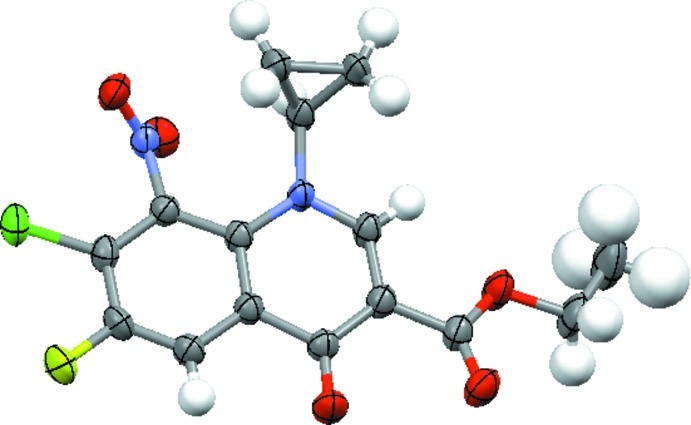
Molecular structure of the title compound. The thermal ellipsoids are drawn at the 30% probability level.

**Fig. 2.**
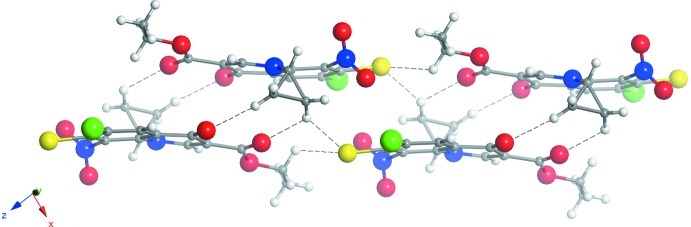
Molecular packing displaying C–H···Cl and C–H···O interactions in the title compound (I).

## supplementary crystallographic information

### Comment

Fluoroquinolone derivatives have been widely investigated as drugs against bacterial infections. Ciprofloxacine, one derivative of fluoroquinolone, represents one of the most effective antiinfectious drugs currently in clinical use (Li *et al.*, 2000; Mitscher 2005). In the present paper, we describe the title compound, I, which has been synthesized from 2,4,-di chloro-5-fluoro-3-nitrobenzoic acid according to the published literature (Al-Hiari *et al.*, 2006) and (Al-Qawasmeh *et al.*, 2009). The title compound is an important synthetic intermediate for the synthesis of the analogues of the antimicrobial drug ciprofloxcaine. The title molecule crystallizes in the centrosymmetric triclinic space group P-1. In the crystal structure of (I), the molecules are held together by C—H···O [3.065 (5)-3.537 (5) Å] and C—H···Cl [3.431 (5)-3.735 (4) Å] (Table 1).

### Experimental

The title compound was synthesized according to the published literature (Al-Hiari *et al.*, 2006) and it has been recrystallized from hot ethanol to produce a yellow crystalline material

### Refinement

Refinement of F^2^ against ALL reflections. The weighted R-factor wR and goodness of fit S are based on F^2^, conventional R-factors R are based on F, with F set to zero for negative F^2^. The threshold expression of F^2^ > σ(F^2^) is used only for calculating R-factors(gt) etc.and is not relevant to the choice of reflections for refinement. R-factors based on F^2^ are statistically about twice as large as those based on F, and R- factors based on ALL data will be even larger. The H atom was located from difference Fourier syntheses and its position and isotropic displacement parameter refined freely.

### Crystal data

**Table:**

C_15_H_12_ClFN_2_O_5_	*F*(000) = 364
*M**_r_* = 354.72	none
Triclinic, *P*1	*D*_x_ = 1.533 Mg m^−^^3^
Hall symbol: -P 1	Melting point: 438 K
*a* = 8.2339 (16) Å	Mo *K*α radiation, λ = 0.71073 Å
*b* = 9.1523 (18) Å	Cell parameters from 1074 reflections
*c* = 10.736 (2) Å	θ = 3.1–29.0°
α = 85.60 (3)°	µ = 0.29 mm^−^^1^
β = 81.20 (3)°	*T* = 291 K
γ = 74.13 (3)°	Needle, yellow
*V* = 768.5 (3) Å^3^	0.96 × 0.35 × 0.21 mm
*Z* = 2	

### Data collection

**Table:**

Oxford Diffraction Xcalibur Eos diffractometer	2713 independent reflections
Radiation source: Enhance (Mo) X-ray Source	1617 reflections with *I* > 2σ(*I*)
Graphite monochromator	*R*_int_ = 0.031
Detector resolution: 16.0534 pixels mm^-1^	θ_max_ = 25.0°, θ_min_ = 3.1°
ω scans	*h* = −8→9
Absorption correction: multi-scan (*CrysAlis PRO*; Oxford Diffraction, 2009)	*k* = −8→10
*T*_min_ = 0.857, *T*_max_ = 1.000	*l* = −12→10
4468 measured reflections	

### Refinement

**Table:**

Refinement on *F*^2^	Secondary atom site location: difference Fourier map
Least-squares matrix: full	Hydrogen site location: inferred from neighbouring sites
*R*[*F*^2^ > 2σ(*F*^2^)] = 0.060	H-atom parameters constrained
*wR*(*F*^2^) = 0.195	*w* = 1/[σ^2^(*F*_o_^2^) + (0.0824*P*)^2^ + 0.0377*P*] where *P* = (*F*_o_^2^ + 2*F*_c_^2^)/3
*S* = 1.05	(Δ/σ)_max_ < 0.001
2713 reflections	Δρ_max_ = 0.29 e Å^−^^3^
218 parameters	Δρ_min_ = −0.26 e Å^−^^3^
0 restraints	Extinction correction: *SHELXL97* (Sheldrick, 2008), Fc^*^=kFc[1+0.001xFc^2^λ^3^/sin(2θ)]^-1/4^
Primary atom site location: structure-invariant direct methods	Extinction coefficient: 0.013 (5)

### Special details

**Table:**

Experimental. CrysAlisPro, Agilent Technologies, Version 1.171.35.11 Empirical absorption correction using spherical harmonics, implemented in SCALE3 ABSPACK scaling algorithm. (CrysAlisPro; Oxford Diffraction, 2009)
Geometry. All esds (except the esd in the dihedral angle between two l.s. planes) are estimated using the full covariance matrix. The cell esds are taken into account individually in the estimation of esds in distances, angles and torsion angles; correlations between esds in cell parameters are only used when they are defined by crystal symmetry. An approximate (isotropic) treatment of cell esds is used for estimating esds involving l.s. planes.
Refinement. Refinement of *F*^2^ against ALL reflections. The weighted *R*-factor *wR* and goodness of fit *S* are based on *F*^2^, conventional *R*-factors *R* are based on *F*, with *F* set to zero for negative *F*^2^. The threshold expression of *F*^2^ > σ(*F*^2^) is used only for calculating *R*-factors(gt) *etc*.and is not relevant to the choice of reflections for refinement. *R*-factors based on *F*^2^ are statistically about twice as large as those based on *F*, and *R*- factors based on ALL data will be even larger. The H atom was located from difference Fourier syntheses and its position and isotropic displacement parameter refined freely.

### Fractional atomic coordinates and isotropic or equivalent isotropic displacement parameters (Å^2^)

**Table:**

	*x*	*y*	*z*	*U*_iso_*/*U*_eq_	
Cl1	0.12355 (14)	0.02366 (11)	0.15569 (10)	0.0800 (4)	
N2	0.4807 (4)	−0.2165 (3)	−0.2558 (3)	0.0577 (8)	
F1	0.1920 (3)	0.2933 (2)	0.0257 (2)	0.0921 (8)	
N1	0.2887 (5)	−0.2409 (3)	0.0032 (3)	0.0651 (8)	
O1	0.5691 (4)	0.1923 (3)	−0.3780 (3)	0.0833 (9)	
C1	0.3081 (4)	−0.0978 (3)	−0.0616 (3)	0.0571 (9)	
O3	0.1470 (4)	−0.2615 (3)	0.0265 (3)	0.0833 (9)	
C3	0.2613 (5)	0.1687 (4)	−0.0426 (4)	0.0663 (10)	
O5	0.8693 (4)	−0.2246 (3)	−0.5300 (3)	0.0892 (10)	
O4	0.8073 (4)	0.0240 (3)	−0.5725 (3)	0.0872 (9)	
C7	0.5977 (5)	−0.2042 (4)	−0.3557 (3)	0.0616 (10)	
H7A	0.6527	−0.2921	−0.3992	0.074*	
O2	0.4183 (4)	−0.3294 (3)	0.0328 (3)	0.0830 (9)	
C2	0.2348 (4)	0.0321 (4)	0.0076 (3)	0.0611 (9)	
C5	0.4362 (4)	0.0483 (4)	−0.2238 (3)	0.0572 (9)	
C9	0.5534 (5)	0.0667 (4)	−0.3412 (4)	0.0607 (9)	
C6	0.4075 (4)	−0.0917 (3)	−0.1800 (3)	0.0544 (9)	
C8	0.6428 (5)	−0.0756 (4)	−0.3988 (3)	0.0615 (10)	
C10	0.7786 (5)	−0.0812 (4)	−0.5092 (4)	0.0673 (10)	
C15	0.4290 (5)	−0.3592 (4)	−0.2387 (4)	0.0669 (11)	
H15A	0.4878	−0.4359	−0.1803	0.080*	
C4	0.3601 (5)	0.1772 (4)	−0.1551 (4)	0.0668 (11)	
H4A	0.3768	0.2704	−0.1862	0.080*	
C14	0.3847 (5)	−0.4180 (4)	−0.3509 (4)	0.0797 (13)	
H14A	0.4181	−0.5271	−0.3605	0.096*	
H14B	0.3885	−0.3580	−0.4292	0.096*	
C13	0.2480 (5)	−0.3550 (4)	−0.2451 (4)	0.0774 (12)	
H13A	0.1693	−0.2570	−0.2595	0.093*	
H13B	0.1989	−0.4262	−0.1907	0.093*	
C11	1.0073 (6)	−0.2448 (5)	−0.6353 (5)	0.1002 (16)	
H11A	0.9628	−0.2012	−0.7124	0.120*	
H11B	1.0908	−0.1937	−0.6197	0.120*	
C12	1.0855 (8)	−0.4041 (6)	−0.6479 (6)	0.146 (3)	
H12A	1.1749	−0.4193	−0.7182	0.219*	
H12B	1.0016	−0.4540	−0.6617	0.219*	
H12C	1.1322	−0.4457	−0.5722	0.219*	

### Atomic displacement parameters (Å^2^)

**Table:**

	*U*^11^	*U*^22^	*U*^33^	*U*^12^	*U*^13^	*U*^23^
Cl1	0.0797 (8)	0.0847 (8)	0.0611 (7)	−0.0052 (6)	0.0105 (5)	−0.0156 (5)
N2	0.0693 (19)	0.0411 (14)	0.0518 (19)	−0.0083 (13)	0.0134 (15)	−0.0013 (12)
F1	0.0937 (18)	0.0661 (13)	0.104 (2)	−0.0100 (12)	0.0191 (15)	−0.0361 (12)
N1	0.075 (2)	0.0571 (18)	0.053 (2)	−0.0090 (17)	0.0059 (17)	−0.0042 (14)
O1	0.107 (2)	0.0508 (14)	0.083 (2)	−0.0210 (14)	0.0142 (17)	0.0036 (13)
C1	0.057 (2)	0.0463 (18)	0.061 (2)	−0.0079 (16)	0.0012 (18)	−0.0004 (16)
O3	0.080 (2)	0.0817 (18)	0.079 (2)	−0.0246 (16)	0.0223 (16)	−0.0017 (15)
C3	0.071 (2)	0.0511 (19)	0.071 (3)	−0.0066 (18)	0.001 (2)	−0.0195 (18)
O5	0.084 (2)	0.0671 (16)	0.091 (2)	−0.0053 (15)	0.0381 (17)	−0.0004 (15)
O4	0.089 (2)	0.0769 (17)	0.084 (2)	−0.0202 (16)	0.0145 (17)	0.0147 (15)
C7	0.066 (2)	0.0482 (18)	0.056 (2)	−0.0011 (17)	0.0079 (19)	0.0019 (16)
O2	0.093 (2)	0.0639 (15)	0.080 (2)	−0.0013 (15)	−0.0148 (17)	0.0056 (14)
C2	0.054 (2)	0.063 (2)	0.057 (2)	−0.0038 (17)	0.0024 (18)	−0.0075 (17)
C5	0.061 (2)	0.0502 (18)	0.055 (2)	−0.0107 (16)	0.0004 (18)	−0.0015 (16)
C9	0.066 (2)	0.0518 (19)	0.059 (2)	−0.0126 (17)	−0.0004 (19)	0.0021 (16)
C6	0.055 (2)	0.0463 (18)	0.054 (2)	−0.0047 (15)	0.0003 (17)	−0.0010 (15)
C8	0.064 (2)	0.056 (2)	0.057 (2)	−0.0111 (17)	0.0043 (19)	0.0020 (16)
C10	0.067 (2)	0.068 (2)	0.058 (3)	−0.012 (2)	0.0045 (19)	0.0027 (19)
C15	0.080 (3)	0.0407 (18)	0.063 (3)	−0.0031 (18)	0.017 (2)	0.0002 (16)
C4	0.074 (3)	0.0480 (19)	0.071 (3)	−0.0110 (18)	0.004 (2)	−0.0055 (17)
C14	0.109 (3)	0.0484 (19)	0.071 (3)	−0.021 (2)	0.027 (2)	−0.0162 (18)
C13	0.081 (3)	0.057 (2)	0.084 (3)	−0.017 (2)	0.023 (2)	−0.0202 (19)
C11	0.081 (3)	0.094 (3)	0.096 (4)	−0.007 (3)	0.047 (3)	0.001 (3)
C12	0.147 (5)	0.108 (4)	0.131 (5)	0.004 (4)	0.075 (4)	−0.005 (4)

### Geometric parameters (Å, º)

**Table:**

Cl1—C2	1.716 (4)	C5—C6	1.399 (4)
N2—C7	1.347 (4)	C5—C9	1.494 (5)
N2—C6	1.396 (4)	C9—C8	1.442 (5)
N2—C15	1.471 (4)	C8—C10	1.494 (5)
F1—C3	1.345 (4)	C15—C14	1.487 (5)
N1—O3	1.218 (4)	C15—C13	1.492 (5)
N1—O2	1.220 (4)	C15—H15A	0.9800
N1—C1	1.471 (4)	C4—H4A	0.9300
O1—C9	1.221 (4)	C14—C13	1.498 (5)
C1—C2	1.391 (5)	C14—H14A	0.9700
C1—C6	1.408 (5)	C14—H14B	0.9700
C3—C4	1.358 (5)	C13—H13A	0.9700
C3—C2	1.382 (5)	C13—H13B	0.9700
O5—C10	1.336 (5)	C11—C12	1.431 (7)
O5—C11	1.459 (5)	C11—H11A	0.9700
O4—C10	1.192 (4)	C11—H11B	0.9700
C7—C8	1.357 (4)	C12—H12A	0.9600
C7—H7A	0.9300	C12—H12B	0.9600
C5—C4	1.384 (5)	C12—H12C	0.9600
			
C7—N2—C6	118.9 (3)	N2—C15—C14	117.5 (3)
C7—N2—C15	117.2 (3)	N2—C15—C13	119.2 (3)
C6—N2—C15	123.7 (3)	C14—C15—C13	60.4 (3)
O3—N1—O2	124.7 (3)	N2—C15—H15A	116.1
O3—N1—C1	119.0 (3)	C14—C15—H15A	116.1
O2—N1—C1	116.3 (3)	C13—C15—H15A	116.1
C2—C1—C6	121.3 (3)	C3—C4—C5	120.6 (3)
C2—C1—N1	115.2 (3)	C3—C4—H4A	119.7
C6—C1—N1	123.2 (3)	C5—C4—H4A	119.7
F1—C3—C4	120.4 (3)	C15—C14—C13	60.0 (3)
F1—C3—C2	118.0 (3)	C15—C14—H14A	117.8
C4—C3—C2	121.6 (3)	C13—C14—H14A	117.8
C10—O5—C11	115.8 (3)	C15—C14—H14B	117.8
N2—C7—C8	126.1 (3)	C13—C14—H14B	117.8
N2—C7—H7A	116.9	H14A—C14—H14B	114.9
C8—C7—H7A	116.9	C15—C13—C14	59.7 (2)
C3—C2—C1	118.4 (3)	C15—C13—H13A	117.8
C3—C2—Cl1	120.0 (3)	C14—C13—H13A	117.8
C1—C2—Cl1	121.5 (3)	C15—C13—H13B	117.8
C4—C5—C6	120.2 (3)	C14—C13—H13B	117.8
C4—C5—C9	116.7 (3)	H13A—C13—H13B	114.9
C6—C5—C9	123.1 (3)	C12—C11—O5	108.5 (4)
O1—C9—C8	126.4 (3)	C12—C11—H11A	110.0
O1—C9—C5	120.5 (3)	O5—C11—H11A	110.0
C8—C9—C5	113.1 (3)	C12—C11—H11B	110.0
N2—C6—C5	117.9 (3)	O5—C11—H11B	110.0
N2—C6—C1	124.2 (3)	H11A—C11—H11B	108.4
C5—C6—C1	117.9 (3)	C11—C12—H12A	109.5
C7—C8—C9	119.6 (3)	C11—C12—H12B	109.5
C7—C8—C10	119.8 (3)	H12A—C12—H12B	109.5
C9—C8—C10	120.6 (3)	C11—C12—H12C	109.5
O4—C10—O5	122.6 (4)	H12A—C12—H12C	109.5
O4—C10—C8	126.8 (4)	H12B—C12—H12C	109.5
O5—C10—C8	110.6 (3)		

### Hydrogen-bond geometry (Å, º)

**Table:**

*D*—H···*A*	*D*—H	H···*A*	*D*···*A*	*D*—H···*A*
C14—H14*A*···O1^i^	0.97	2.54	3.489 (4)	167
C14—H14*B*···O1^ii^	0.97	2.51	3.471 (5)	172
C15—H15*A*···O2^iii^	0.98	2.58	3.537 (5)	165
C4—H4*A*···O2^iv^	0.93	2.71	3.065 (5)	104
C13—H13*A*···O4^ii^	0.97	2.71	3.439 (5)	132
C11—H11*A*···Cl1^v^	0.97	2.91	3.431 (5)	115
C13—H13*A*···Cl1^vi^	0.97	2.89	3.735 (4)	146

Symmetry codes: (i) *x*, *y*−1, *z*; (ii) −*x*+1, −*y*, −*z*−1; (iii) −*x*+1, −*y*−1, −*z*; (iv) −*x*+1, −*y*, −*z*; (v) *x*+1, *y*, *z*−1; (vi) −*x*, −*y*, −*z*.
